# Effect of tumor shape and size on drug delivery to solid tumors

**DOI:** 10.1186/1754-1611-6-4

**Published:** 2012-04-25

**Authors:** M Soltani, Pu Chen

**Affiliations:** 1 Waterloo Institute for Nanotechnology, Department of Chemical Engineering, University of Waterloo, Waterloo, ON., Canada N2L 3G1

**Keywords:** Drug delivery, Solid tumors, Computational fluid dynamics, Tumor shape and size, Interstitial fluid

## Abstract

Tumor shape and size effect on drug delivery to solid tumors are studied, based on the application of the governing equations for fluid flow, i.e., the conservation laws for mass and momentum, to physiological systems containing solid tumors. The discretized form of the governing equations, with appropriate boundary conditions, is developed for predefined tumor geometries. The governing equations are solved using a numerical method, the element-based finite volume method. Interstitial fluid pressure and velocity are used to show the details of drug delivery in a solid tumor, under an assumption that drug particles flow with the interstitial fluid. Drug delivery problems have been most extensively researched in spherical tumors, which have been the simplest to examine with the analytical methods. With our numerical method, however, more complex shapes of the tumor can be studied. The numerical model of fluid flow in solid tumors previously introduced by our group is further developed to incorporate and investigate non-spherical tumors such as prolate and oblate ones. Also the effects of the surface area per unit volume of the tissue, vascular and interstitial hydraulic conductivity on drug delivery are investigated.

## Introduction

Cancer causes one in every four deaths in North America and is the second most common cause of death worldwide [1]. Solid tumors account for 85% of human cancers [1]. Although the most important cancer treatment is surgical removal of such tumors, the key to a successful cure often involves efficient delivery of anticancer drugs to the tumor site after the surgery. Many new drugs have been developed, but lack of an efficient means of delivery makes them less effective. Moreover, some of these drugs induce biochemical reactions in the body that produce toxicity. Bioengineers are primarily concerned with both the transport of drugs within the body (which usually involves systemic delivery through the blood supply) and with biochemical reactions or conversions at tumor sites [2,3]. All of these problems demonstrate that solutions to drug delivery limitations are urgently needed [1].

Two factors inhibit the effective delivery of drugs within tumors: non-uniform blood supply and non-uniform interstitial pressure distribution [4,5]. Drugs concentrate most readily in areas with the best blood supply. In solid tumors, these areas are closest to the blood vessels (the vasculature) and a tumor’s peripheral walls; however, 90% of a tumor receives little or no drug, meaning that treated tumors tend to regrow, as only their outer cells have been killed by the drug [6-9]. Variations in the interstitial (i.e., of connective tissue in which cells are embedded) pressure reduce fluid exchange and further inhibit the movement of the drug into the center of the tumor.

**Figure 1:**
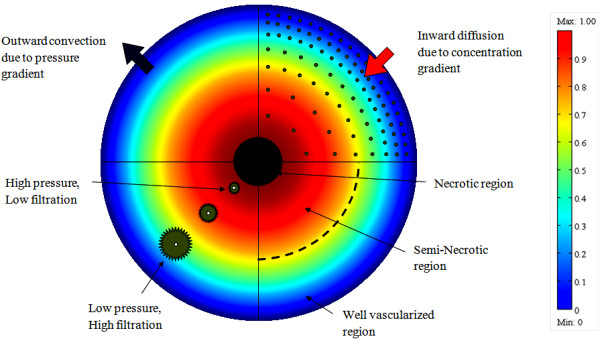
**Cross sectional schematic of a solid tumor that shows the three different regions of a solid tumor, IFP distribution, drug concentration and filtration distribution from blood vessels**[14]**, doi: 10.1371/journal.pone.0020344.g001.**

Previous research has shown that drugs administered systemically are not uniformly reaching tumor sites. Baxter et al. have shown that, in addition to blood flow heterogeneities and impeded interstitial transport, another mechanism effectively contributes to the non-uniform distribution of drugs: high interstitial pressure in solid tumors [10-12]. High interstitial pressure limits drug transport in two ways: 1) it reduces the driving force that is the result of the interstitial fluid pressure and vascular pressure difference; 2) it moves fluid to the outer layers of the tumor in which the interstitial pressure has its minimum. Both these effects are shown schematically in Figure 1. The first effect decreases the driving force for transcapillary exchange of fluid and, therefore, drugs. Low filtration (liquid source per tissue volume) occurs at the center of the tumor as a result of the high interstitial pressure or low driving force, and high filtration occurs at the periphery of the tumor as a result of the low interstitial pressure or high driving force. The second effect of the high interstitial pressure results in a radially outward convective flux in the interstitium as fluid flows towards the outer layers of the tumor. This effect is illustrated in Figure 1 as an outward convection due to interstitial fluid pressure gradient. The value of the radially outward fluid velocity at the tumor rim for a solid tumor (mammary adenocarcinoma s.c.) with a 1 cm radius, 4.2 g , is 0.1∼0.2*μm*/*s*[2]. Figure 1 demonstrates another important feature of drug delivery to tumors: an inward diffusion due to concentration gradient of the drug. Effective penetration into a solid tumor requires that the velocity of the diffusion process be higher than that of the convection process [13]. On the other hand, uniformly distributed high interstitial pressure in the center of a tumor blocks convection and, consequently, causes the heterogeneous perfusion of blood into the center of tumors, resulting in heterogeneous distribution of drugs [12].

Modeling drug delivery involves processes such as drug diffusion, convective transport in extracellular matrices, drug extravasation from blood vessels, tissue elimination by the lymphatic system, and intracellular internalization. In all of these processes, computational fluid dynamics (CFD) can play a crucial role in clarifying the mechanisms of drug delivery from the injection site to absorption by a solid tumor. In-vitro release profiles of systemically administered drugs have been combined with state of the art computational fluid dynamics simulations to predict both temporal and spatial drug delivery in many studies [15-20]. Temporal and spatial changes in blood flow have also been studied with a focus on capillary-network or single-vessels [21,22]. Before Baxter et al. [2,23,24] introduced their innovative model of interstitial pressure as a function of tumor radius, little was known about tumor modeling, except that interstitial pressure is highest at the center of a tumor [25] and that pressure is directly proportional to tumor size [25,26]. The main focus of future drug delivery modeling involves the transport of the drug in tissues after drug release by either systemic administration or implantation.

As spherical tumors are the easiest to examine analytically, they have been used in most studies. The effect of tumor shape has not been addressed in the literature except the analytical study of by El-Kareh and Secomb [27]. The numerical method introduced here allows modeling of more complex shapes and promotes a better understanding of the complex mechanisms of interstitial fluid transport that effective drug delivery must depend on. In studying tumor modeling, the numerical method, which introduces more features of drug delivery to solid tumors, is more effective than the analytical method. To design an optimum scheme for drug delivery, the transport mechanisms and obstacles to drug delivery have to be clarified, which is one of the main objectives of this paper.

The proposed CFD model is made for both spherical and non-spherical tumors and their surrounding normal tissues. This model can be further extended to study geometries reconstructed from high resolution images. In this study the tumor and its surrounding tissue are assumed to be rigid porous media. The vasculature as a source term varies spatially. The grid generation divides the whole domain or geometry into finite volumes, called meshes. Interstitial fluid flow equations in porous media are solved using a CFD code (based on the proposed CFD model) that employs unstructured grids (tetrahedral elements) to handle non-spherical tumors.

## Methods

The tissues most relevant to this discussion are the vasculature (vessels that, with the heart, comprise the circulatory system that carries blood throughout the body), the interstitium (or interstitial space), and the cells. Also relevant is the lymphatic system, which, simply put, is responsible for tissue drainage. The vasculature system involves vessels (essentially tube-like structures) of varying sizes, from the large arteries and veins down to the much smaller arterioles, venules and capillaries [28]. The interstitium is formed of fibers such as collagen, which gives structural stability, glycosaminoglycans (GAG), and other proteins. Together, these fibers make up the gel-like region between blood vessels and cells. The cells, occupying the cellular space, include specific tissue cells (i.e., the cancer cells of a solid tumor) and others such as pericytes, macrophages, and fibroblasts not discussed in this paper [2,23,24]. In normal physiology, fluid seeps slowly but constantly from blood vessels into the surrounding tissues. The lymphatic system then reabsorbs this lost fluid and returns it to the blood stream. All the above tissues must be considered in any discussion of drug delivery to tumors. Specifically, to be effective, drugs must cross the blood vessel wall, traverse the interstitial space containing the cancer cells, and bind to and (if the target is intracellular) penetrate the cancer cell membrane. The lymphatic system then removes excess fluid and debris. However, a lack of such lymphatic drainage in solid tumors has been reported in the literature [2,3]. Computer simulations show that this lack of lymphatic system involvement may result in a build up of interstitial pressure, leading to cessation of the usual blood seepage from vessels. Consequently, large molecules cannot be carried out of vessels to interact with tissue. As some drug particles, including Monoclonal Antibodies (MAbs) used to fight cancer, are large and move very slowly within tissues, they cannot reach the tumor site and are thus ineffective [2].

### Mathematical Model of Interstitial Fluid Transport

The distribution of vasculature and cells in solid tumors is spatially heterogeneous. Solid tumors have a center necrotic core where most of the cells are dead. The outer boundary of such tumors contains many exchange vessels, a large blood supply, and fast-dividing cells. Therefore, the mathematical model should be accurate enough to include the dependency of physiological parameters, such as the hydraulic conductivity, on space; that is, it must be able to clearly represent all the physical variations in a tumor. Nevertheless, because the time scale of transport phenomena is much less than that of tumor growth, the physiological parameters can be considered time independent [2]. In a macroscopic model, only the distribution of variables, such as interstitial pressure and concentration, over the length scale of the tumor radius is important, and microscopic characteristics, such as blood vessels, cells, and the interstitial matrix, are not involved directly. Comparison of the tumor radius, on the order of magnitude of 1*cm*, *O*(1*cm*), with the intercapillary distance (the average distance between two capillaries), *O*(100*μm*), indicates that variations over microscopic length scales can be averaged out [29]. The screening length, $\sqrt{\mathrm{\mu k}}$ (in which *μ*and *k* are the viscosity of the interstitial fluid and the hydraulic conductivity of the interstitium, respectively), is on the order of 60*Å*; therefore, the fluid transport in the tumor interstitium can be described by Darcy’s law for flow through a porous medium [3,29-33]:

(1) $$\begin{array}{cc} v=-k\nabla P_{i} & \;\text{in general}\\ v=-k\frac{\partial P_{i}}{\mathrm{\partial r}} & \;\text{for axisymmetric flow} \end{array}$$

where $k\left[cm^{2}/\mathrm{mmHg}s\right]$$P_{i}\left[\mathrm{mmHg}\right]$$v\left[m/s\right]$and *rcm* are the hydraulic conductivity of the interstitium, the interstitial fluid pressure, the interstitial fluid velocity and the radial position, respectively. In the case of anisotropic and heterogeneous porous media, *k* is a tensor and function of the location in the medium.

The mass balance equation for a steady state incompressible fluid shows that the divergence of the fluid is zero, or mathematically,

(2) ∇·v=0

The same equation is also applicable in porous media if there is no fluid source or fluid sink in the medium. However, in most biological tissues, sources and sinks are present. For instance, between interstitial space and the blood or lymph vessels, fluid is exchanged; therefore, the steady state incompressible form of the continuity equation must be modified as

(3) $$\nabla \cdot v=\phi_{B}(r)-\phi_{L}(r)$$

where **v** is the fluid velocity in the representative elementary volume (REV). The continuity equation can also be written as

(4) $$\nabla \cdot (\varepsilon v_{f})=\left\{\begin{array}{c} \phi_{B}(r)-\phi_{L}(r)\;\text{for}\;r\geq R_{n}\\ 0\;\;\;\;\text{for}\;r<R_{n} \end{array}\right)$$

where _*Rn*_*cm*, *ε*, _**v***f*_*m*/*s*, _*ϕB*_(*r*)^*s*−1^, and _*ϕL*_(*r*)^*s*−1^ are the radius of the necrotic core, the porosity or the volume fraction of fluid, the fluid velocity averaged in the volume of the fluid phase, the fluid source term, and the lymphatic drainage term, respectively. In biological tissues, the two last terms signify the rate of fluid flow per unit volume from blood vessels into the interstitial space and from the interstitial space into lymph vessels, respectively. Both rates can be evaluated through Starling’s law. It should be noted that Eq. (4) in this general form is applicable to any kind of biological tissue, whether normal or cancerous. In dead tissues, with no flow in the blood or lymph vessels, the value for both terms is zero. The fluid source term is governed by Starling’s law as follows [34,35]:

(5) $$\phi_{B}(r)=\frac{J_{V}}{V}=\frac{L_{P}S}{V}(P_{B}-P_{i}-\sigma_{s}(\Pi_{B}-\Pi_{i}))$$

**Figure 2:**
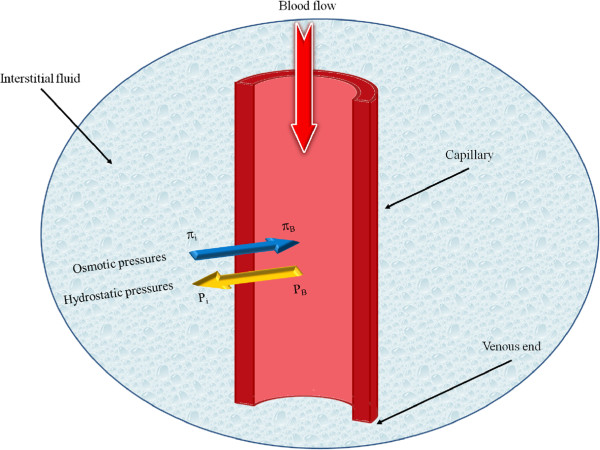
**Capillary microcirculation schematic and different types of pressure**[14]**, doi:10.1371/journal.pone.0020344.g002.**

The parameters used in Eq. (5) are $\frac{J_{V}}{V}\left[s^{-1}\right]$ , the volumetric flow rate out of the vasculature per unit volume of tissue (liquid source per tissue volume or filtration flux); $\frac{S}{V}\left[cm^{-1}\right]$, the surface area per unit volume for transport in the tumor; $L_{p}\left[\frac{\mathrm{cm}}{\mathrm{mmHgs}}\right]$ , the hydraulic conductivity of the microvascular wall; $P_{B}\left[\mathrm{mmHg}\right]$, the vascular pressure; _*σs*_, the average osmotic reflection coefficient for plasma proteins; $\Pi_{B}\left[\mathrm{mmHg}\right]$, the osmotic pressure of the plasma; and $\Pi_{i}\left[\mathrm{mmHg}\right]$, the osmotic pressure of the interstitial fluid. The different types of pressure used in Eq. (5) are shown in Figure 2. It should be noted that the lymphatic drainage term is proportional to the pressure difference between the interstitium and the lymphatics:

(6) $$\begin{array}{c} \phi_{L}(r)=\frac{J_{L}}{V}=\frac{L_{\mathrm{PL}}S_{L}}{V}(P_{i}-P_{L})\;\text{for}\;r\geq R_{n}\\ \phi_{B}(r)=\phi_{L}(r)=0\;\;\;\;\;\;\text{for}\;r<R_{n} \end{array}$$

The parameters used in these equations are $\frac{J_{L}}{V}\left[s^{-1}\right]$, the volumetric flow rate into the lymphatics; $\frac{S_{L}}{V}\left[cm^{-1}\right]$, the surface area per unit volume of the lymphatics; $L_{\mathrm{pL}}\left[\frac{\mathrm{cm}}{\mathrm{mmHgs}}\right]$, the hydraulic conductivity of the lymphatic wall; and $P_{L}\left[\mathrm{mmHg}\right]$, the hydrostatic pressure of the lymphatics.

Combination of Darcy’s law and the continuity equation results in

(7) $$-\nabla \cdot k\nabla P_{i}=\phi_{B}(r)-\phi_{L}(r)$$

For a very special case, when *k* is constant and there are no sinks and sources (for example, in the necrotic core), the interstitial pressure can be expressed by the very well-known Laplace equation.

(8) $$\nabla^{2}P_{i}=0$$

If all parameters except *P*_*i*_ are assumed to be constant, substituting Eqs. (5) and (6) in Eq. (7) results in

(9) $$-k\nabla^{2}P_{i}=\frac{L_{P}S}{V}(P_{B}-P_{i}-\sigma_{s}(\Pi_{B}-\Pi_{i}))-\frac{L_{\mathrm{PL}}S_{L}}{V}(P_{i}-P_{L})$$

Rearranging Eq. (9) for an arbitrary shape of a solid tumor results in

(10) $$\nabla^{2}P_{i}=\frac{L_{P}S+L_{\mathrm{PL}}S_{L}}{\mathrm{kV}}(P_{i}-P_{\mathrm{ss}})$$

Equation (10) for a spherical solid tumor with radius *R* is written as,

(11) $$\nabla^{2}P_{i}=\frac{\alpha^{2}}{R^{2}}(P_{i}-P_{\mathrm{ss}})$$

_*PSS*_ is defined later by Eq. (15). Using the definition of the Laplace operator, Eq. (12), in the spherical coordinate system, Eq. (11) is written as in Eq. (13).

(12) $$\begin{array}{cc} \Delta & =\nabla^{2}=\frac{1}{r^{2}}\frac{\partial }{\mathrm{\partial r}}\left(r^{2}\frac{\partial }{\mathrm{\partial r}}\right)+\frac{1}{r^{2}\sin\theta }\frac{\partial }{\partial \theta }\left(\sin\theta \frac{\partial }{\partial \theta }\right)\\ \;+\frac{1}{r^{2}\overset{2}{\sin}\theta }\frac{\partial^{2}}{\partial^{2}\phi } \end{array}$$

(13) $$\frac{1}{r^{2}}\frac{\partial }{\mathrm{\partial r}}\left(r^{2}\frac{\partial P_{i}}{\mathrm{\partial r}}\right)=\frac{\alpha^{2}}{R^{2}}(P_{i}-P_{\mathrm{ss}})$$

In Eqs. (11) and (13), the ratio of interstitial resistance to vascular resistance is introduced in terms of *α*, the dimensionless parameter defined by Eq. (14).

(14) $$\alpha =R\sqrt{(L_{P}S+L_{\mathrm{PL}}S_{L})/\mathrm{kV}}$$

(15) $$P_{\mathrm{SS}}=(L_{P}SP_{e}+L_{\mathrm{PL}}S_{L}P_{L})/(L_{P}S+L_{\mathrm{PL}}S_{L})$$

The steady state pressure, _*PSS*_, is the interstitial pressure at which the efflux from the vasculature and influx into the lymphatics are equal, and is defined by Eq. (15). Effective pressure, *P*_*e*_, in Eq. (15), is a parameter defined by vascular pressure, plasma osmotic pressure, and interstitial osmotic pressure through Eq. (16).

(16) $$P_{e}=P_{B}-\sigma_{s}(\Pi_{B}-\Pi_{i})$$

Applying the appropriate boundary conditions and also all the constants mentioned earlier, the more general form of the governing equation, Eq. (10), can be used to calculate the interstitial fluid velocity (IFV) and interstitial fluid pressure (IFP) profiles in solid tumors. Of note, for spherical solid tumors, simpler forms of the governing equation, Eq. (11) or (13), can be used. Additionally, *α* is a dimensionless constant used here for convenience (only in spherical tumors); in general (arbitrary shapes), more fundamental physical constants such as *L*_*p*_, *S*, *L*_*PL*_, *S*_*L*_, *k*, and *V *, used in Eq. (10), should be used. No lymph vessels in a solid tumor means *S*_*L*_ = 0; thus, Eqs. (10) , (13) and (14) can be simplified as follows:

(17) $$\nabla^{2}P_{i}=\frac{L_{P}S}{\mathrm{kV}}(P_{i}-P_{e})$$

(18) $$\frac{1}{r^{2}}\frac{\partial }{\mathrm{\partial r}}\left(r^{2}\frac{\partial P_{i}}{\mathrm{\partial r}}\right)=\frac{\alpha^{2}}{R^{2}}(P_{i}-P_{e})$$

(19) $$\alpha =R\sqrt{(L_{P}S)/\mathrm{kV}}$$

in which the interstitial pressure that yields zero net volume flux out of the vasculature is called the effective pressure, *P*_*e*_, Eq. (16). The steady state pressure and effective pressure in solid tumors with no lymph vessels are the same. If *P*_*i*_ = *P*_*e*_, no exchange of fluid occurs between the interstitial space and blood vessels.

Due to symmetry, there is a no flux boundary condition at the center of the tumor; i.e.,

(20) $$\nabla P_{i}=0\;\mathrm{or}\;\frac{\partial P_{i}}{\mathrm{\partial r}}=0\;\text{for}\;\;r=0$$

At the outer edge of the solid tumor, (*r* = *R* for a spherical tumor), two types of boundary conditions are possible. In the first type, where the pressure in the surrounding tissue is fixed, the tumor pressure at the outer edge is the same as the surrounding pressure, *P*_*sur*_.

(21) $$P_{i}=P_{\mathrm{sur}}\;\text{for outer region,}\;\;r\in \Omega$$

**Figure 3:**
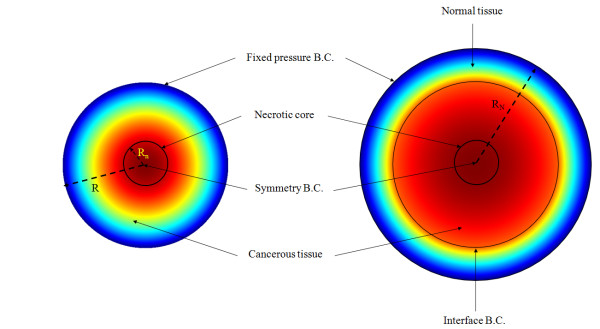
**Two types of boundary conditions at the outer edge of the tissue**[14]**, doi:10.1371/journal.pone.0020344.g003.**

This condition is applicable for an isolated tumor [36,37]. In the second type, the solid tumor is surrounded by normal tissues. Pressure decreases smoothly over a distance; therefore, the continuity of pressure and velocity should be considered as an appropriate boundary condition for this case as the following conditions occur simultaneously:

(22) $${\left(-k_{t}\frac{dP_{i}}{\mathrm{dr}}\right|}_{\Omega^{-}}={\left(-k_{n}\frac{dP_{i}}{\mathrm{dr}}\right|}_{\Omega^{+}}$$

(23) $${\left(P_{i}\right|}_{\Omega^{-}}={\left(P_{i}\right|}_{\Omega^{+}}$$

where ^*Ω*−^and ^*Ω* + ^indicate the tumor and normal tissue at the outer edge of the solid tumor; _*kt*_ and _*kn*_ are the hydraulic conductivity of the interstitium in tumor and normal tissues, respectively. It should be noted that, in the second type, all the equations mentioned for the tumor tissue have to be solved for the normal tissue as well. For the normal tissue, far enough from the solid tumor that the pressure is constant, the first type of boundary condition, Eq. (21), must be applied. Figure 3 shows these two types of boundary conditions. The solution can now be obtained analytically or numerically to find the IFV and IFP profiles for each of the two boundary conditions. In this work, the numerical method has been used. An element-based finite volume method (EB-FVM) is applied to discretize the equations. The EB-FVM combines the capability of the finite element method (FEM) for handling complex geometries with the sound physical-based properties of the finite volume method (FVM) [38]. The discretized form of the governing equations, in their general form, is then linearized and solved implicitly. The Semi Implicit Method for Pressure Linked Equations (SIMPLE) algorithm is used as the coupling method for pressure and velocity terms. Finally, the converged form of the solution is calculated using an iterative method. To improve the convergence rate, the method of successive over-relaxation (SOR) is applied, with an under relaxation factor equal to 0.75. The criterion for the convergence is to reduce the residual by 6 orders of magnitudes. In order to check the grid independency of the code, the largest tumor in this study is chosen and the results for three different grids are compared, indicating the conservative property of the numerical method. The final choice of the grid in this test case includes 11904 control volumes. For other tumor geometries the same mesh parameters are applied.

The material properties for tumor and normal tissue were taken from the simulation studies of Jain and Baxter [12] and are shown in Table 1. It should be noted that tissue properties vary greatly among different organs, for both normal and cancerous tissues; therefore, parameters introduced in Table 1 should be updated for new applications. As mentioned earlier, tissue transport properties are often anisotropic and heterogeneous. Geometric and physiological properties of anisotropic and heterogeneous tissues affect drug delivery. This issue can be solved with the help of diffusion tensor imaging (DTI). A good application of this method in brain tumors is discussed by Linninger et al. [39].

**Table 1:** **Material properties used in numerical simulations, as taken from [**[12]**]**

**Parameter**	**Tissue**	**Baseline value**	**Reference**
$L_{p}\left[\mathrm{cm}/\mathrm{mmHgs}\right]$	Normal	0.36×1^0−7^	Rippe et al. (1978)
	Tumor	2.80×1^0−7^	Jain (1987a)
$k\left[cm^{2}/\mathrm{mmHgs}\right]$	Normal	8.53×1^0−9^	Swabb et al. (1974)
	Tumor	4.13×1^0−8^	Jain (1987a)
$S/V\left[cm^{-1}\right]$	Normal	70	Pappenheimer et al. (1951)
	Tumor	200	Hilmas and Gilette (1974)
$P_{B}\left[\mathrm{mmHg}\right]$	Both	15.6	Brace and Guyton (1977)
$\Pi_{B}\left[\mathrm{mmHg}\right]$	Both	20	Brace and Guyton (1977)
$\Pi_{i}\left[\mathrm{mmHg}\right]$	Normal	10	Wiederhielm (1979)
	Tumor	15	Jain (1987a)
_*σs*_	Normal	0.91	Ballard and perl (1978)
	Tumor	0.82	Curry (1984)

## Results

**Figure 4:**
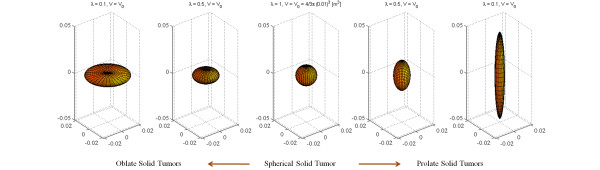
Different shapes of solid tumors: spherical, oblate, and prolate.

The actual tumor shape is not necessarily spherical. How to handle the patient’s specific tumor and tissue dimensions in transport equations is discussed in the literature [40]. To study different shapes of solid tumors – spherical, oblate (flattened), and prolate (elongated) – shown in Figure 4, the governing equation is solved numerically [14]. Generally speaking, it is believed that increasing the hydraulic conductivity of tumor vessels increases the drug delivery to tumor cells. Many researchers, such as Sands et al. [7], Khawli et al. [41], LeBerthon et al. [42], and Cope et al. [43], have stated the abovementioned hypothesis and tried to explain their experimental results based on it.

**Figure 5:**
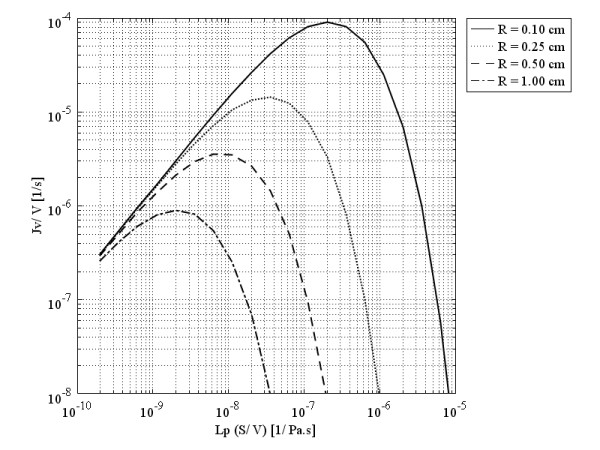
Liquid source (volumetric flow rate out of the vasculature per unit volume of tissue or filtration flux) per tissue volume at the tumors’ center, as a function of multiplication of hydraulic conductivity of the microvascular wall and surface area per unit volume.

Figure 5 shows the volumetric flow rate out of the vasculature per unit volume, also called liquid source per tissue volume or filtration flux, at the different tumors’ centers as a function of the multiplication of hydraulic conductivity of the microvascular wall and surface area per unit volume, based on Eq. (5), which can be rewritten as Eq. (24):

(24) $$\phi_{B}(r)=\frac{J_{V}}{V}=\frac{L_{P}S}{V}(P_{e}-P_{i})$$

The parameters used in Eq. (24) are $\frac{J_{V}}{V}\left[s^{-1}\right]$ , the volumetric flow rate out of the vasculature per unit volume of tissue; $\frac{S}{V}\left[cm^{-1}\right]$ , the surface area per unit volume for transport in the tumor; $L_{p}\left[\frac{\mathrm{cm}}{\mathrm{mmHgs}}\right]$ , the hydraulic conductivity of the microvascular wall; $P_{e}\left[\mathrm{mmHg}\right]$, the effective pressure; and $P_{i}\left[\mathrm{mmHg}\right]$, the interstitial fluid pressure. All parameters used to solve this equation are listed in Table 1.

Figure 5, shows that in a spherical solid tumor with a certain radius, *R*, if hydraulic conductivity is increased, the volumetric flow rate first increases to reach an optimum value (maximum) and then decreases. This graph also indicates that an increase in the diameter of spherical solid tumors causes a decrease in the optimum value of the volumetric flow rate. Decreasing one order of magnitude of tumor radius increases the volumetric flow rate approximately two orders of magnitude. This optimum value occurs in response to a small value of hydraulic conductivity in spherical solid tumors with a relatively larger diameter. The values of hydraulic conductivity that result in optimum values of the flux are called optimum values of hydraulic conductivity. The filtration flux (or the volumetric flow rate), shown in Figure 5 as a function of *L*_*p*_*S*/*V*, indicates that the usual values for *L*_*p*_ or *S*/*V* in the literature, listed in Table 1, are much greater than the optimum values shown in this graph. $L_{p}^{\mathrm{opt}}$, the optimum value of hydraulic conductivity, can be found easily in a graph such as Figure 5 for different tumor sizes, or one can find the appropriate size for a specific tumor tissue with a known value of *L*_*p*_ to have the maximum drug flow rate. In Figure 5, *L*_*p*_ changes linearly from its minimum values to its maximum values. On the other hand, the pressure difference (between effective pressure and IFP) changes from *P*_*e*_ (when IFP is equal to zero) to zero (when IFP is equal to the effective pressure). When *L*_*p*_ is at its minimum, the pressure difference is at its maximum, and vice versa. This circumstance results in a peak in the volumetric flow rate curve in terms of the hydraulic conductivity of the microvascular wall, *L*_*p*_, and surface area per unit volume, *S*/*V*, or a combination of these factors through Eq. (24).

**Figure 6:**
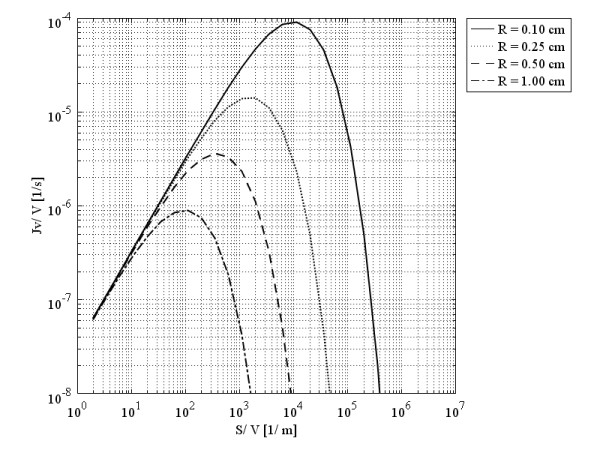
Liquid source per tissue volume at the tumors’ center, as a function of surface area per unit volume.

Baxter et al. [12] stated that, with a decrease in the surface area of the tumor vasculature, the amount of fluid filtered and therefore the amount of drug filtered decreases as well. However, Figure 6 shows that this statement is true for just part of the curves. The same behavior occurs for surface area per unit volume instead of hydraulic conductivity; the graph showing this similarity is shown in Figure 6.

**Figure 7:**
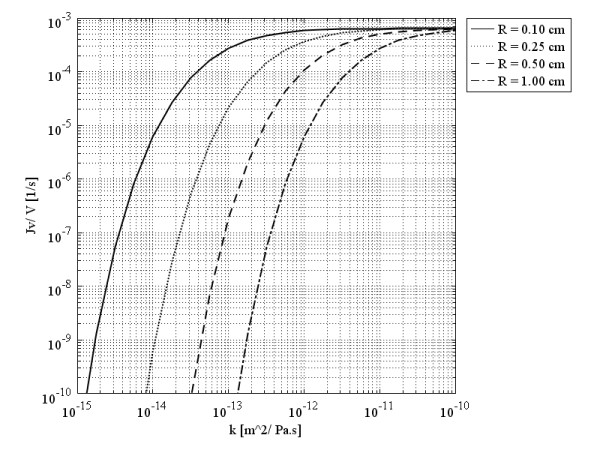
Liquid source per tissue volume at tumors’ center, as a function of the hydraulic conductivity of the interstitium.

For most cancerous tissues, the hydraulic conductivity of the interstitium is not reported accurately in the literature [27]. For those cases reported in the literature, such as recent work of Linninger et al. [44] on estimating the hydraulic conductivity in porous brain tissue, one can use inverse method and apply numerical method presented in this study to calculate the hydraulic conductivity and compare it with the estimated value in [44]. However, the change in *k* has been studied here, and the result, shown in Figure 7, indicates that *k* and *L*_*p*_ have opposite effects on filtration flux. Figure 7 shows the behavior of volumetric flow rate as a function of the hydraulic conductivity of the interstitium at the centers of spherical tumors of different radii. This figure demonstrates that in smaller tumors (0.1 cm radius in this case) the maximum value of the volumetric flow rate occurs in a smaller value of the hydraulic conductivity of the interstitium, at least two orders of magnitude less than that of the larger tumor with a 1 cm-sized radius. In fact, increasing the value of *k*, decreases the amount of *α*. Low amounts of *α*result in low values of IFP. Based on Eq. (24), the effect of low IFP values is an increase in the volumetric flow rate.

**Figure 8:**
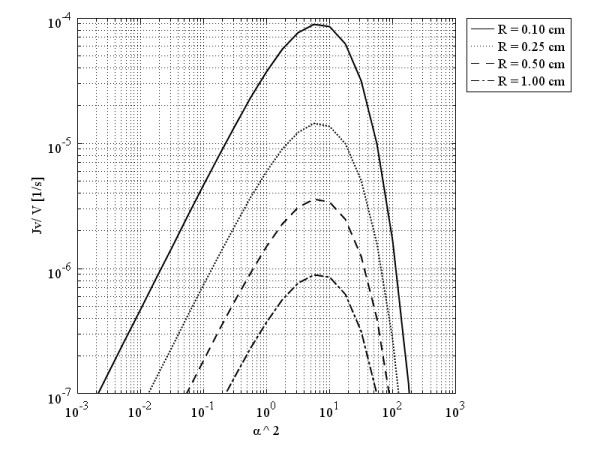
**Liquid source per tissue volume at tumors’ center, as a function of ***α***, a dimensionless parameter.**

**Figure 9:**
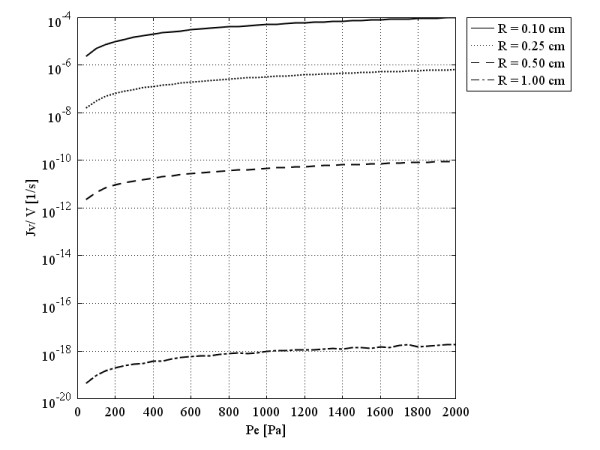
Liquid source per tissue volume at tumors’ center, as a function of effective pressure.

Figure 8 shows that in a spherical solid tumor of a certain diameter, with an increase in *α*, which is the dimensionless parameter defined based on Eq. (19), the volumetric flow rate increases first, but after reaching an optimum value (maximum), it decreases. The interesting point about this graph is that the optimum values of the volumetric flow rate for different sizes of spherical solid tumors occur at the same value of *α*. This figure demonstrates that increasing one order of magnitude of tumor radius decreases the volumetric flow rate approximately two orders of magnitude at the same value of ^*α*2^, almost equal to 6. The simultaneous effect of effective pressure, *P*_*e*_, and size is shown in Figure 9. The pattern for different sizes is the same; the more the effective pressure, the more the volumetric flow rate. As discussed before [14] and shown in Figure 9, the lower the tumor size, the lower the IFP, which results in a more volumetric flow rate.

**Figure 10:**
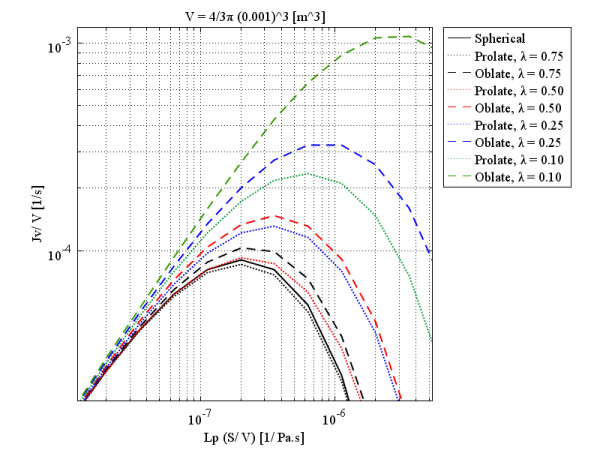
Liquid source per tissue volume at the center of different tumor shapes with the same volume as a spherical 0.1 cm radius tumor, as a function of multiplication of hydraulic conductivity of the microvascular wall and surface area per unit volume.

**Figure 11:**
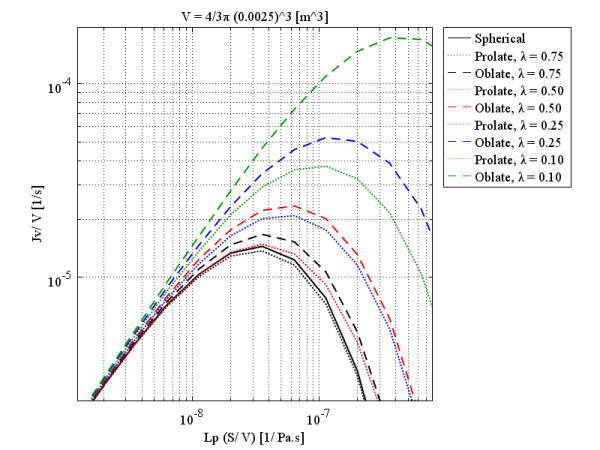
Liquid source per tissue volume at the center of different tumor shapes with the same volume as a spherical 0.25 cm radius tumor, as a function of multiplication of hydraulic conductivity of the microvascular wall and surface area per unit volume.

**Figure 12:**
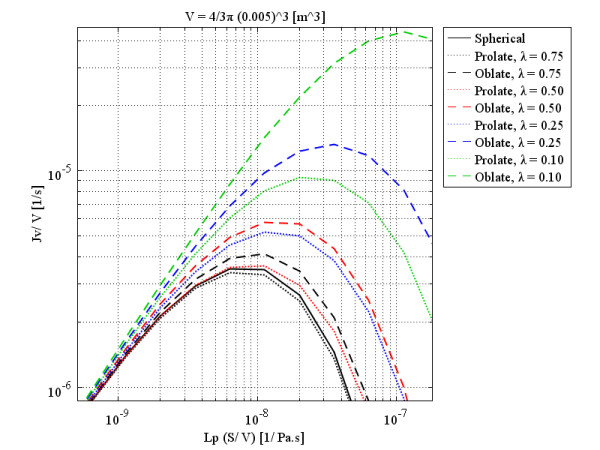
Liquid source per tissue volume at the center of different tumor shapes with the same volume as a spherical 0.5 cm radius tumor, as a function of multiplication of hydraulic conductivity of the microvascular wall and surface area per unit volume.

**Figure 13:**
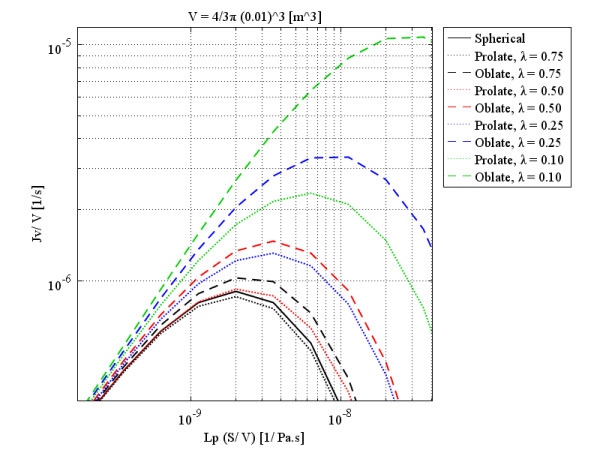
Liquid source per tissue volume at the center of different tumor shapes with the same volume as a spherical 1.0 cm radius tumor, as a function of multiplication of hydraulic conductivity of the microvascular wall and surface area per unit volume.

Real tumors have unusual shapes. To show the effect of tumor shape and size on *P*_*i*_ distribution and also the filtration flux for different values of *L*_*p*_ and *S*/*V*, the study in [27] has been loosely reworked here but with a different approach in terms of formulation and solution method to cover a greater variety of tumor shapes and sizes. As mentioned earlier, the general form of the governing equation has been used in this paper to study non-spherical solid tumors, shapes not covered in the literature. Figure 10 shows volumetric flow rate behavior at the center of different tumor shapes that have the same volume as a spherical 0.1 cm radius tumor, as a function of multiplication of the hydraulic conductivity of the microvascular wall and surface area per unit volume. Different colors show the different values of *λ* (or *a*/*b*), the ratio of minor to major axes of both prolate and oblate spheroids. Clearly, when *λ* is 1, all three shapes have become spherical. As is shown, the general pattern for different shapes (spherical, prolate, and oblate solid tumors) is the same. Rapid increase by the enhancement of hydraulic conductivity or surface area per unit volume to an optimum value (maximum), and then the decrease by enhancement of those parameters, is the main characteristic of Figures 10 to 13. Simultaneous effects of shape and size of the tumors on the flux (volumetric flow rate) distribution can be explained according to these four figures.

**Figure 14:**
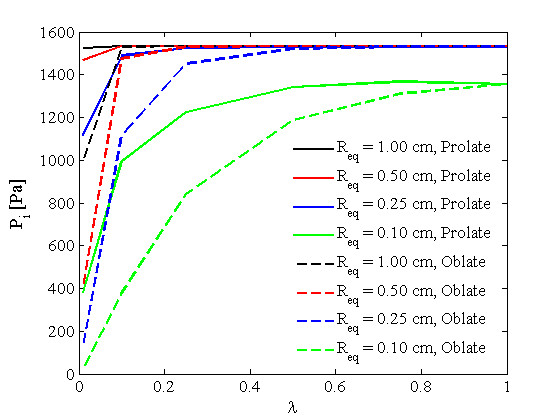
**Pressure distribution at the center of different tumor shapes, as a function of minor to major axes ratio. ***R*_*eq*_ is the radius of a spherical tumor with the same volume as both prolate and oblate tumors.

As the labels indicate, each figure is for a specific volume, which is the same for all three shapes and is based on the size of spherical shapes. These figures show that although, qualitatively, all three shapes have the same behavior, quantitatively, spherical and prolate solid tumors are very much closer to each other in behavior than they are to that of oblate solid tumors. This statement is truer for small tumors than larger ones and also for smaller values of *λ*. As discussed, by increasing the radius of solid tumors, the order of volumetric flow rate decreases, as through Figures 10 to 13. On the other hand, when *λ* approaches one, all three shapes show the same behavior. Figure 14 shows the interstitial pressure distribution at the tumor center for different values of *λ* (or *a*/*b*). It should be mentioned that for each curve, the volume is constant, and *R*_*eq*_ is the radius of a spherical tumor with the same volume as both prolate and oblate tumors. This figure indicates that, for large tumors, except for extremely low values of *a*/*b*, which occur with very thin shapes of tumors, the central pressure is independent of shape. This study also shows that there are two limits for the central pressure value: for large tumors, *R*_*eq*_ > 0.25 cm, *P*_*i*_ ≈ *P*_*e*_ (especially for prolate solid tumors), and for small tumors, *R*_*eq*_ < 0.02 cm, *P*_*i*_ ≈ *P*_*sur*_, no matter what the shape of the tumor. For sizes between these two limits, for example *R*_*eq*_ = 0.1 cm, as shown in Figure 14, the central pressure is a function of both tumor shape and size. Thus, for 0.02 cm <*R*_*eq*_ < 0.25 cm, *P*_*sur*_ <*P*_*i*_ <*P*_*e*_.

**Figure 15:**
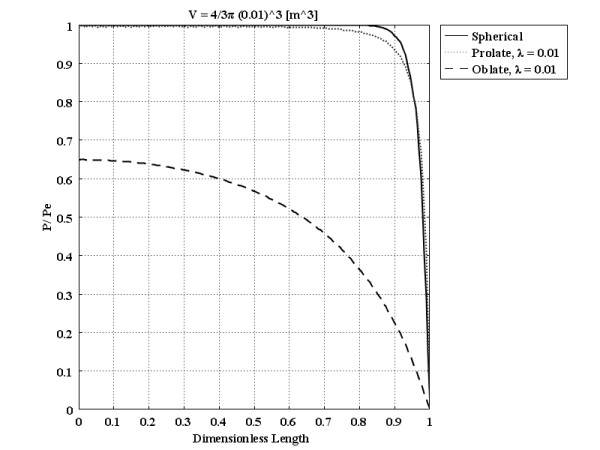
Pressure distribution of different tumor shapes with the same volume as a spherical 1.0 cm radius tumor, as a function of dimensionless length.

Two limiting cases (in which a prolate spheroid approaches cylindrical geometry and the oblate spheroid approaches a planar slab) are interesting to study with more detail. These limiting cases can be achieved by choosing the very low values of *λ* (0.01 in this case). The IFP distributions throughout these geometries for *R*_*eq*_ = 1.0 cm are shown in Figure 15 to specify the effects of size and shape more carefully in these cases. This figure shows that for non-spherical tumors, everything hinges on the smallest dimension, and the other dimension is not important. When the smallest dimension of the prolate tumor is close to or in a comparable size to the spherical one, the pressure distribution is almost the same, as shown in Figure 15. For the oblate tumor, as the order of the smallest dimension is very different from that of the spherical one, pressure distribution is totally different in Figure 15. The same figures as Figure 15 for other sizes of *R*_*eq*_ can be shown here. Only the central IFP values for all *R*_*eq*_ values are shown in Figure 14 when *λ*is very small, 0.01.

## Discussion

The effect of increasing the hydraulic conductivity of microvessels, *L*_*p*_, in terms of drug delivery to solid tumors is significant to this discussion. There are two opposing phenomena: the total fluid filtration flux for the whole solid tumor and local filtration within the solid tumor; therefore, the liquid source term, the filtration flux at the center of the tumor, has its highest value for an optimum value of *L*_*p*_, as shown by El-Kareh and Secomb [27]. As they have shown in their study analytically, and is indicated here numerically, this optimum value of *L*_*p*_ is a function of the size and shape of solid tumors. An increase in *L*_*p*_ has a direct effect on the total rate and an opposite effect on the local filtration flux. Increased vessel leakiness cannot result in more uniform drug distribution through tumors much larger than 0.25 cm.

Starling’s law shows that, because of low diffusivity, the dominant form of drug transport through vessel walls (transvascular transport) is convection [27]. Based on Eq. (5), both hydraulic conductivity of the vessel wall and pressure difference across the wall are effective in convective transport, again according to Starling’s law. Similar behavior is true for *S*/*V*; thus, an increase in *L*_*p*_ and *S*/*V* does not have a positive effect, and there are optimum values for both of these parameters. However, the analysis of the work done in [27], as well as this study, shows that, except when the nodule radius is smaller than 0.1 cm, the advantage of decreasing *P*_*i*_ is more important than that of decreasing the surface area.

As indicated, an increase in the hydraulic conductivity results in a volumetric flow increase, but after a continued increase, volumetric flow approaches a certain value for all different tumors. This pattern for tumors of all diameters is the same; the only difference is that the smaller the tumor diameter, the sooner the leveling in volumetric flow.

The parameter *α*does not apply exactly in a non-spherical tumor; therefore, the general form of the governing equation, different from the more specific form of that applied in spherical solid tumors, has to be used here. This parameter, *α*, is a combination of other fundamental variables such as hydraulic conductivity, which turns out to have a clean short definition when solved for a sphere. This study shows that for a distinctly non-spherical tumor (such as a prolate or oblate), everything hinges on the shortest dimension, and the longer dimension is irrelevant, as any fluid or material flows to reach the closest surface.

Because El-Kareh et al. [27] used an analytical approach to solve the governing equations in prolate and oblate spheroidal shapes, they had to change the coordinate system from a spherical one to a more complicated coordinate system, with oblate and prolate spheroidal coordinates. In this coordinate system, the governing equation, the Helmholtz equation, has a much more complicated form than the original equation. The solution for this new form of equation is a Fourier series. In the work proposed here, the numerical method is applied and, obviously the solution is not limited to any specific shape, offering a freedom that is one of the main advantages of the numerical method over the previous one.

### Conclusions

The parameter *α*/*R* is independent of tumor geometry. It is a function of hydraulic conductivity (an interstitial property) and vessel permeability. The main problem is defining a characteristic length instead of *R* in non-spherical tumors. The pressure profile, however, is a function of tumor geometry. Using a solution for IFP that was calculated assuming a spherical profile will give a solution as a function of *α*, *r*, and *R*. Not surprisingly, equations tailored to the different tumor shapes will yield different IFPs, as these pressures are the outcomes of the equations, and thus reflect the discrepancies. What will happen, whether a tumor is a sphere or not, is that IFP will be approximately equal to the vascular pressure, *P*_*B*_, throughout most of the interior. Only near the boundary, whether that boundary is for a sphere or spheroid, will there be a steep pressure gradient as IFP falls to the pressure of the surrounding tissue. For a very small radius, the differences will be significant. Above the critical radius, shape is almost irrelevant.

This study shows that, as a rule, it is not true that the leakier the vessels, the higher the value of convective transport of drugs to solid tumors. The results do show that only in spherical solid tumors with a radius of less than 0.25cm, or in spheroidal ones with the same volume, can drug convection be increased by making vessels more leaky. For spheroidal shapes, the convection of drugs inside is higher than it is in spherical ones, and it seems that for more irregular shapes, which are generally found in the body due to limitations imposed by neighboring tissues and organs such as the brain, this effect is more marked.

For shapes studied in this paper, results show that the dependency of the maximized flux (as a function of *L*_*p*_ and *S*/*V* or their multiplication) on size is much stronger than its dependency on shape. It should be mentioned that due to very low diffusivity, the high permeability of vessels cannot support homogeneous distribution inside tumors because this high value results in more diffusive transport only in a narrow area around the vessels.

## Competing interest

The authors declare that they have no competing interests.

## Author’s contribution

Conceived and designed the experiments: MS PC. Performed the experiments: MS. Analyzed the data: MS PC. Wrote the paper: MS PC. Both authors read and approved the final manuscript.
